# Phlebosclerotic Colitis – An Enigma Among Ischemic Colitis

**DOI:** 10.25259/JCIS-30-2019

**Published:** 2019-05-24

**Authors:** Rishi Philip Mathew, Safwat Girgis, Malcolm Wells, Gavin Low

**Affiliations:** 1 Departments of Radiology and Diagnostic Imaging, Division of Gastroenterology, University of Alberta, 8440 112 St NW, Edmonton, Canada.; 2 Departments of Laboratory Medicine and Pathology, Division of Gastroenterology, University of Alberta, 8440 112 St NW, Edmonton, Canada.; 3 Department of Medicine, Division of Gastroenterology, University of Alberta, 8440 112 St NW, Edmonton, Canada.

**Keywords:** Idiopathic mesenteric phlebosclerosis, Ischemic colitis, Mesenteric vein calcification, Phlebosclerotic colitis

## Abstract

Phlebosclerotic Colitis is a rare, potentially life-threatening condition of unclear etio-pathogenesis seen almost exclusively in Asians and people of Asian descent. The condition predominantly affects the right hemicolon and imaging plays a crucial role in its diagnosis. Here we report the only second documented case of phlebosclerotic colitis in North America in a 60-year-old Canadian resident of Vietnamese descent with a history of consuming herbal medication (*sanshishi*) in soup for 2-3 decades.

## INTRODUCTION

Phlebosclerotic colitis (PC) also known as idiopathic mesenteric phlebosclerosis is a rare form of ischemic colitis. The first case was brought to the attention of the medical community at a Japanese meeting in 1989 by Iwashita *et al.* and the term “PC” was first coined by Yao *et al.* in 2000.^[1,2]^ Since then, fewer than 80 cases have been documented in the English medical literature.^[3]^ PC is almost exclusively seen in the Asian population (Japan, China, Korea, and Taiwan) or those with Asian ancestry. Unlike other forms of ischemic colitis which are primarily caused by arterial obstruction due to arteriosclerosis, thrombosis, or embolism, PC is a rare potentially life-threatening ischemic colitis that develops as a result of mesenteric venous calcification and calcification of the affected colonic submucosa. As biopsy results can be insufficient and even non-diagnostic, knowledge of its characteristic imaging pattern and characteristic endoscopic appearance can help make a diagnosis.^[4]^ We present the second documented case of PC in North America, the first being a Canadian resident of Taiwanese descent, reported in 2004.^[5]^

## CASE REPORT

A 60-year-old woman, a resident of Canada and Vietnamese descent, presented for colonoscopy following a fecal immunochemical test (FIT) positive result for cancer screening. She gave a history of recent onset (3–4 months) of intermittent abdominal pain with loose stools up to 3–4 times per day with no nocturnal symptoms and no bright blood per rectum. She gave a history of consuming herbal medication (containing *sanshishi*) in soup for the past 20–30 years. She is a non-smoker and teetotaler. Her family history was unremarkable, and she had never been on or consumed prescription medications. She had no known allergies, and on physical examination, her abdomen was soft, non-tender with no hepatosplenomegaly.

**Figure 1:**
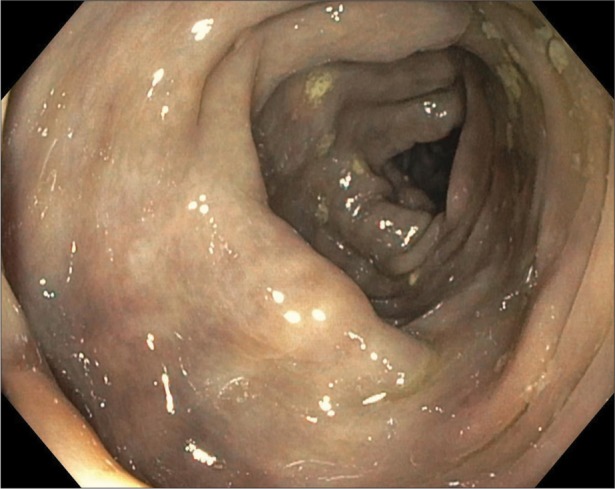
A 60-year-old female with fecal immunochemical test positive and recent onset (3–4 months) of intermittent abdominal pain with loose stools. Colonoscopy image at the splenic flexure showing a dusky and friable appearing colon.

As the patient was FIT positive, a colonoscopy was performed. On colonoscopy, the rectum appeared normal. At the rectosigmoid junction, the mucosa of the sigmoid colon appeared purple in color with congested veins. At the splenic flexure, the colon was noted to appear friable with contact bleeding and appeared dusky [Figure 1], progressively worsening while traversing through the transverse colon into the ascending colon and cecum. Appearances were suggestive of ischemic colitis. Scattered ulcerations were noted in the cecum. The terminal ileum was intubated up to 15 cm and appeared endoscopically normal. Endoscopic-guided biopsies revealed a firm hardened mucosa. The biopsies were sent for histopathology analysis.

**Figure 2:**
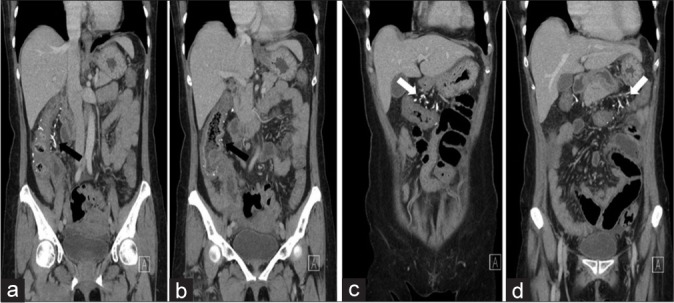
A 60-year-old female with fecal immunochemical test positive and recent onset (3–4 months) of intermittent abdominal pain with loose stools. Coronal reformatted computed tomography images showing colonic wall thickening involving the cecum, ascending colon (a and b), and transverse colon (c) up to the splenic flexure (d), with characteristic threadlike or serpentine calcified subserosal and mesenteric veins (arrows) in the distribution along these bowel loops.

**Figure 3:**
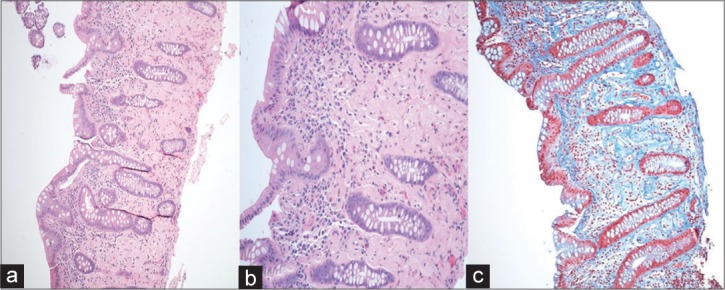
A 60-year-old female with fecal immunochemical test positive and recent onset (3–4 months) of intermittent abdominal pain with loose stools. Histopathological images of the ascending colon shot at ×100 and ×200 routine ×200 (routine H and E stains) (a and b), and ×100 for the Masson’s trichrome stain (c) showing diffuse crypt distortion with patchy lymphoplasma eosinophilic infiltrate, crypt atrophy, and moderate degree of crypt loss. The surface epithelium shows mild damage. There are marked lamina propria hyalinization and fibrosis as clearly highlighted in the Masson’s trichrome stain (c). All these features are in keeping with chronic ischemic changes that are consistent with phlebosclerotic colitis.

As there were features suspicious of ischemic colitis on colonoscopy, a computed tomography angiography (CTA) of the abdomen [Figure 2a-d] was performed which revealed colonic wall thickening of the ascending colon and transverse colon, along with characteristic threadlike or serpentine calcified subserosal and mesenteric veins in the distribution of the cecum and ascending and transverse colon. However, the arterial vasculature was normal. Mesenteric fat stranding was also noted in the affected pericolonic region. The rest of the large bowel was unremarkable. On histopathology [Figure 3a-c], biopsy fragments of the colonic mucosa showed diffuse crypt distortion with patchy lymphoplasma eosinophilic infiltrate, crypt atrophy, and moderate degree of crypt loss. The surface epithelium showed mild damage. There were marked lamina propria hyalinization and fibrosis highlighted in the Masson’s trichrome stain. Findings were in keeping with chronic ischemic changes that were consistent with PC.

**Figure 4:**
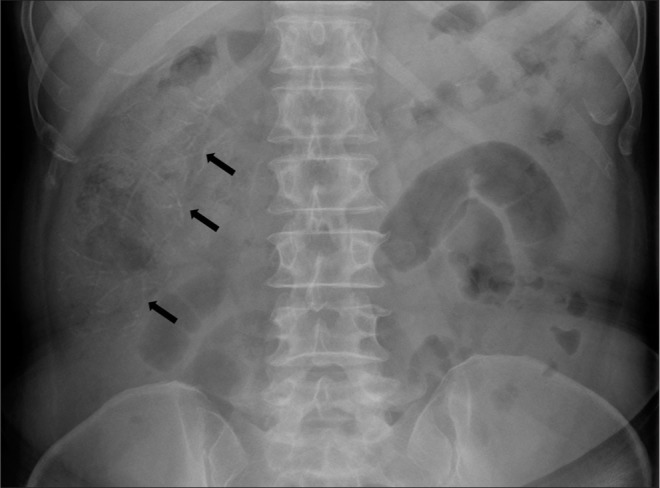
A 60-year-old female with fecal immunochemical test positive and recent onset (3–4 months) of intermittent abdominal pain with loose stools. Magnified abdominal radiograph showing faint threadlike linear calcifications (arrows) oriented along the ascending colon.

Three months later, the patient presented with abdominal pain, for which an abdominal radiography was done. Careful examination of the abdominal radiograph [Figure 4] revealed linear threadlike calcifications oriented along the ascending colon. Inflammatory changes in the form of mucosal edema were noted in the transverse colon. No features of bowel obstruction were noted. Air was noted up to the rectum.

On repeat colonoscopy, a demarcation of the normal colon and the area of dark discolouration was identified at 30 cm from the anal verge, and this was marked with a tattoo. The patient was discussed at multidisciplinary meeting for consideration of anticoagulation versus future surgery.

## DISCUSSION

Patients with PC presented with non-specific symptoms such as diarrhea, nausea, vomiting, abdominal pain, and tarry stool. Some patients in the early stage of the disease can even be asymptomatic, while those with advanced disease can present with intestinal obstruction and even perforation.^[4,6]^ The pathogenesis of PC is not clearly understood. However, in majority of the documented cases of PC, it is believed that ingested biochemicals and toxins, especially herbal medicines, invigorant water of unknown contents, and alcohol have been associated with the disease.^[3,6,7]^ The use of herbal medicine containing *sanshishi* as an ingredient has been proven to be strongly associated with PC.^[8]^

PC primarily affects the right hemicolon, and a proposed hypothesis for this is that the ingested toxins and biochemicals are mostly absorbed from the ascending colon, and chronic stasis within the lumen leads to cephalad migration and along with increased intraluminal pressure causes long-term reduced venous return and ultimately impaired venous drainage and hemorrhagic infarction of the affected colonic wall.^[7,9]^ Few cases have been documented where the pathology extends to involve the transverse colon or even the entire colon. One proven case involved only the left colon, leaving the right colon unaffected.^[9]^

PC has a characteristic endoscopic and radiological appearance that can help diagnose the condition when biopsy is inconclusive. On colonoscopy, the affected colonic mucosa has a characteristic dark purple discoloration. Other colonoscopic findings include erythema, mucosal edema, erosion, ulceration, narrowing of the colonic lumen, loss of haustrations, rigidity of the colonic wall, and focal nodular surface.^[4,10]^ However, some patients can have a negative colonoscopy.^[10]^ The most striking and characteristic finding on abdominal radiography and CT are tortuous threadlike or serpentine mesenteric venous calcifications involving the marginal veins, vena recti, and intramural tributaries that are perpendicularly oriented to the long axis of the affected colonic wall. In the initial stage of the disease, calcifications first involve the peripheral mesenteric veins, followed by the intramural tributaries of mostly the right hemicolon along with sclerosis of the colonic muscular wall. Calcifications of the proximal SMV and inferior mesenteric vein generally have been described in the advanced stage of the disease affecting the entire colon.^[4,7,10]^ In addition to the calcifications, other CT findings include colonic wall thickening and adjacent mesenteric fat stranding. Although CT is the imaging modality of choice for diagnosing PC and for its follow-up, modalities such as barium enema and CTA can also be used for its evaluation. Typical findings on barium enema include luminal narrowing, colonic wall thickening, effacement of the haustral folds, and a “thumbprinting” appearance.^[5,11]^ On venography, dilatation of the veins may be seen along the vasa recta with a decreased venous perfusion leading to under perfusion of the affected bowel.^[5,9,11]^

Management of PC is mostly conservative with close follow-up. Surgery (hemicolectomy and subtotal or total colostomy) is reserved for those patients with severe complications (e.g., intestinal obstruction, perforation, and hemorrhage) and for those with persistent symptoms following conservative management.^[4,12]^ Differential diagnosis for PC includes conditions that cause colonic wall thickening with calcifications such as mucinous adenocarcinoma and leiomyosarcoma. Other causes of chronic mesenteric ischemia such as Churg-Strauss syndrome, Behcet’s disease, lymphocytic phlebitis, Degos disease, Wegener’s granulomatosis, and polyarteritis nodosa do not cause fibrosis or calcifications in the veins.^[3,12]^

## CONCLUSION

PC is a rare bowel pathology of unclear pathogenesis mostly observed in Asians and those with Asian ancestry. Imaging findings of threadlike or serpentine calcifications along the colon and mesenteric veins on CT and a dark purple-colored colonic mucosa on colonoscopy can help to make the diagnosis. Treatment depends on the severity of the disease, ranging from conservative management to prompt surgical intervention.
